# TGFβ-Responsive Stromal Activation Occurs Early in Serrated Colorectal Carcinogenesis

**DOI:** 10.3390/ijms25094626

**Published:** 2024-04-24

**Authors:** Hideaki Tsumuraya, Hirokazu Okayama, Masanori Katagata, Akira Matsuishi, Satoshi Fukai, Misato Ito, Wataru Sakamoto, Motonobu Saito, Tomoyuki Momma, Shotaro Nakajima, Kosaku Mimura, Koji Kono

**Affiliations:** 1 Department of Gastrointestinal Tract Surgery, Fukushima Medical University School of Medicine, Fukushima 960-1295, Japan; tsumu23@fmu.ac.jp (H.T.); m-ktgt@fmu.ac.jp (M.K.); akimm@fmu.ac.jp (A.M.); sfukai@fmu.ac.jp (S.F.); m-saku12@fmu.ac.jp (M.I.); ws1024@fmu.ac.jp (W.S.); moto@fmu.ac.jp (M.S.); tmomma@fmu.ac.jp (T.M.); shotaro@fmu.ac.jp (S.N.); kmimura@fmu.ac.jp (K.M.); kojikono@fmu.ac.jp (K.K.); 2 Department of Multidisciplinary Treatment of Cancer and Regional Medical Support, Fukushima Medical University School of Medicine, Fukushima 960-1295, Japan; 3 Department of Blood Transfusion and Transplantation Immunology, Fukushima Medical University School of Medicine, Fukushima 960-1295, Japan

**Keywords:** colorectal cancer, serrated pathway, TGFβ, serrated lesion, *BRAF* mutation

## Abstract

Activated TGFβ signaling in the tumor microenvironment, which occurs independently of epithelial cancer cells, has emerged as a key driver of tumor progression in late-stage colorectal cancer (CRC). This study aimed to elucidate the contribution of TGFβ-activated stroma to serrated carcinogenesis, representing approximately 25% of CRCs and often characterized by oncogenic *BRAF* mutations. We used a transcriptional signature developed based on TGFβ-responsive, stroma-specific genes to infer TGFβ-dependent stromal activation and conducted in silico analyses in 3 single-cell RNA-seq datasets from a total of 39 CRC samples and 12 bulk transcriptomic datasets consisting of 2014 CRC and 416 precursor samples, of which 33 were serrated lesions. Single-cell analyses validated that the signature was expressed specifically by stromal cells, effectively excluding transcriptional signals derived from epithelial cells. We found that the signature was upregulated during malignant transformation and cancer progression, and it was particularly enriched in CRCs with mutant *BRAF* compared to wild-type counterparts. Furthermore, across four independent precursor datasets, serrated lesions exhibited significantly higher levels of TGFβ-responsive stromal activation compared to conventional adenomas. This large-scale analysis suggests that TGFβ-dependent stromal activation occurs early in serrated carcinogenesis. Our study provides novel insights into the molecular mechanisms underlying CRC development via the serrated pathway.

## 1. Introduction

Colorectal cancer (CRC) develops via the accumulation of genetic and epigenetic alterations. Many CRCs are thought to arise through adenomatous precursor lesions that originate from the conventional adenoma-carcinoma sequence, accompanied by a step-wise acquisition of mutations along with increased chromosomal instability (CIN) [1,2]. Inactivation of the *APC* tumor suppressor gene, which results in the constitutive activation of the Wnt signaling pathway, is the initiating event of developing conventional adenomas [1,3,4]. Subsequent malignant transformation is driven by mutations in the *KRAS* oncogene, promoting cell proliferation and survival through the RAS-RAF-ERK signaling cascade, as well as a functional loss of the *TP53* tumor suppressor gene, which is involved in cell cycle control [3,4]. Moreover, loss-of-function mutations in the *SMAD4* tumor-suppressing gene encoded on chromosome 18q, which is a key mediator of TGFβ signaling, are involved in the late stages of the conventional pathway [1,5,6]. On the other hand, approximately 25% of CRCs are estimated to arise via an alternative pathway, often called the serrated pathway [7,8,9]. Colorectal serrated lesions are categorized into hyperplastic polyps (HPs), sessile serrated lesions (SSLs), and traditional serrated adenomas (TSAs), according to the latest WHO classification (5th edition, 2019), among which SSLs represent a major precursor for the serrated pathway of CRC [10,11]. In contrast to the conventional pathway, serrated lesions, particularly SSLs, commonly harbor *BRAF* mutations but rarely present *APC* mutations. Oncogenic *BRAF* mutations cause the constitutive stimulation of the RAS-RAF-ERK pathway, which results in the dysregulation of cell proliferation, differentiation, and survival, independent of upstream RAS signaling [3,4]. Additional molecular features associated with the serrated pathway include CpG island methylator phenotype (CIMP) and microsatellite instability (MSI) [8,9]. Notably, recent studies have demonstrated that not only *BRAF* mutations but also the inactivation of *SMAD4* or other genes in the TGFβ pathway have an essential role at early stages of serrated tumorigenesis [9]. Although oncogenic *BRAF* mutations alone are inefficient to drive tumorigenesis in mouse models, genetic deletion of *SMAD4*, or loss of TGFβ receptor signaling in intestinal epithelial cells accelerate the initiation and progression of serrated colon tumorigenesis in these *BRAF*-mutant mouse models [12,13,14].

The role of TGFβ signaling during tumor progression in solid cancers is complex, as its effects can differ dramatically depending on the cell type and the conditions [15,16,17,18,19]. TGFβ is known to have a dual role in CRC [15,16]. In the early stages of tumorigenesis, TGFβ functions as a potent tumor suppressor because it induces apoptosis and cytostasis in healthy and premalignant epithelial cells. During tumor progression, TGFβ may induce an epithelial-to-mesenchymal transition (EMT) in cancer cells, a process through which cancer cells acquire mesenchymal phenotypes associated with invasion and metastasis, while bypassing the tumor-suppressive functions of epithelial TGFβ signaling [15,16]. A gene expression profiling study suggested that serrated precursors develop into mesenchymal CRCs, characterized by the upregulation of genes involved in EMT and the TGFβ pathway component [20]. Moreover, in human organoid cultures of premalignant colorectal lesions, TGFβ induces cell death in conventional adenoma-derived organoids but strongly induces EMT in organoids harboring mutant *BRAF* as a model for early serrated lesions [21]. This indicates that high activity of the TGFβ pathway in epithelial cells can direct serrated precursors to mesenchymal CRCs [21]. Therefore, in the serrated pathway, TGFβ plays a crucial role in the malignant transformation and progression of epithelial premalignant cells, at least in part through the induction of EMT.

Although the role of TGFβ in CRC has been predominantly documented in epithelial cells, many recent studies emphasize that the main target of TGFβ is the stromal component of tumor [22,23,24]. In the later stages of tumorigenesis, tumor-promoting effects of stromal TGFβ signaling can occur independently of epithelial cancer cells, in which TGFβ pathway components, such as *SMAD4* and *TGFΒR2*, are often mutationally inactivated [16]. Transcriptomic studies of CRC highlight that the tumor microenvironment (TME) activated by TGFβ is the key driver of poor clinical and therapeutic outcomes in CRC [23,25,26]. TGFβ induces a pro-metastatic gene program particularly in cancer-associated fibroblasts (CAFs) within the TME [22]. Elevated TGFβ levels, increased TGFβ response signatures, and activated TGFβ signaling in the TME (especially CAFs) are associated with poor prognosis in CRC [22,23,26,27].

Although recent studies have reported that dysregulation of epithelial TGFβ signaling can contribute to malignant transition in *BRAF*-induced serrated tumorigenesis, the impact of stromal TGFβ signaling on the serrated pathway remains unexplored. To address the contribution of the TGFβ-activated TME in the serrated pathway, we conducted in silico analyses using a TGFβ-responsive stromal gene signature across multiple transcriptomic cohorts of *BRAF*-mutant and wild-type CRCs, as well as conventional and serrated precursor lesions.

## 2. Results

### 2.1. TGFβ-Stromal Signature (TBSS) in Single-Cell RNA-Seq Analyses

**Figure 1:**
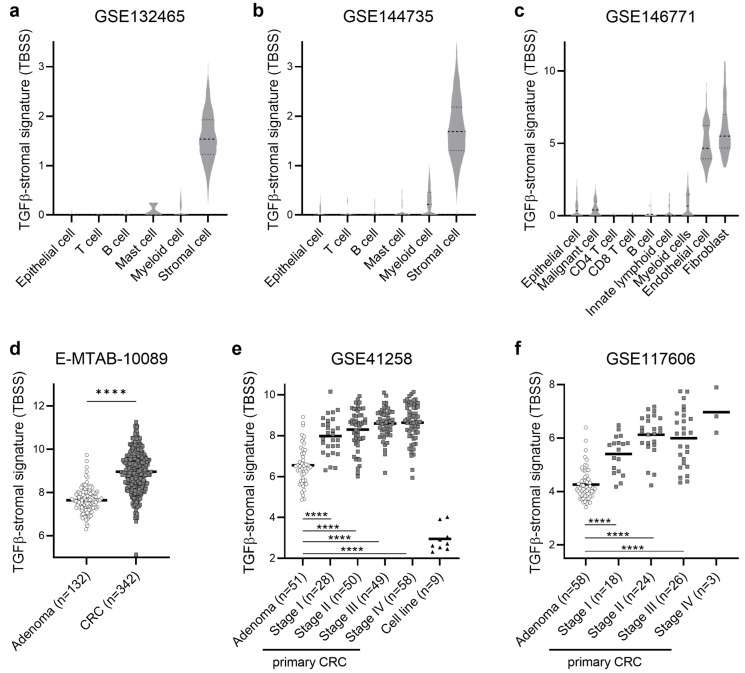
TBSS in single-cell and bulk transcriptomic cohorts of adenomas and CRCs. (**a**–**c**) Validation of the stromal specificity of TBSS in three single-cell RNA-seq datasets of CRC (GSE132465, GSE144735, and GSE146771). (**d**) TBSS levels in 132 conventional adenomas and 342 primary CRCs (E-MTAB-10089). (**e**) TBSS levels in 51 conventional adenomas, 185 primary CRCs at different stages, and 9 cell lines (GSE41258). (**f**) TBSS levels in 58 conventional adenomas and 71 primary CRCs at different stages (GSE117606). **** *p* < 0.0001.

In our recent study, we established the TGFβ-stromal signature (TBSS) using six genes that exhibit specific upregulation in the stromal component in response to TGFβ [28]. These genes were previously identified in our earlier research [27]. Our findings revealed that TBSS was primarily expressed by CAFs and, to a lesser extent, by endothelial cells within the TME, as observed in a limited number of cell-level analyses [28]. We also demonstrated that TBSS in bulk tissues was unrelated to the signals derived from the epithelial component by using transcriptomic CRC datasets of patient-derived organoids, patient-derived xenografts, and cell lines [29]. In the present study, we aimed to further validate the stromal specificity of this signature, and we utilized three independent single-cell RNA-seq datasets of CRC, including GSE132465, GSE144735, and GSE146771 (Supplementary Table S1). We analyzed TBSS levels in approximately 70,000 annotated single cells from a total of 39 primary CRCs. Among six major cell types identified in GSE132465 and GSE144735 [30], TBSS was found to be specific to stromal cells (Figure 1a,b). Correspondingly, Figure 1c shows that TBSS was specifically expressed by fibroblasts and endothelial cells among eight major cell type reported in GSE146771 [31]. This extensive analysis, on the basis of a large number of single-cell RNA-seq data from various cohorts, evidently validated that TBSS originates exclusively from the stromal component of the tumor, with a negligible expression in epithelial cancer cells.

### 2.2. Increased TGFβ-Responsive Stromal Activation during Tumor Progression

We next examined a merged meta-cohort (E-MTAB-10089) comprising bulk microarray data from 132 conventional adenomas and 342 CRCs (Supplementary Table S2) [32]. Our analysis revealed that TBSS was significantly upregulated in CRCs compared to adenomas (Figure 1d). To further validate this finding, we obtained two additional large datasets of conventional adenomas and primary CRCs, representing various stages of the disease, including GSE41258 that consisted of 51 adenomas and 185 CRCs [33], and GSE117606 that consisted of 58 adenomas and 71 CRCs (Supplementary Table S2) [34]. In both cohorts, we confirmed a striking upregulation of TBSS levels in CRCs compared to adenomas. Moreover, we observed a gradual increase in TBSS from stage I to stage IV CRCs (Figure 1e,f). We also noticed that the expression of TBSS was virtually undetectable in the nine CRC cell lines included in GSE41258, further affirming its absence in epithelial cancer cells (Figure 1e).

### 2.3. Serrated and BRAF-Mutant CRCs Exhibited TGFβ-Activated TME

**Figure 2:**
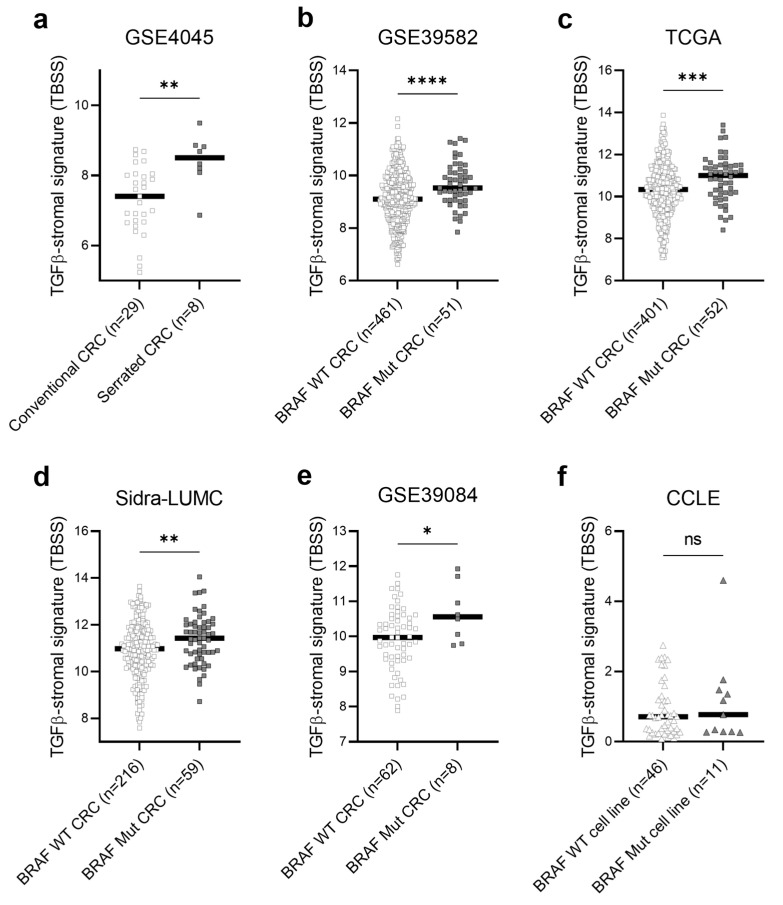
TBSS in multiple cohorts of CRCs. (**a**) TBSS levels in 29 conventional and 8 serrated CRCs (GSE4045). (**b**–**e**) TBSS levels in a total of 1140 *BRAF* wild-type (WT) and 170 *BRAF* mutant (Mut) CRCs in 4 datasets (GSE39582, TCGA, Sidra-LUMC, and GSE39084). (**f**) TBSS levels in 46 *BRAF* WT and 11 *BRAF* Mut CRC cell lines (CCLE). **** *p* < 0.0001, *** *p* < 0.001, ** *p* < 0.01, * *p* < 0.05, ns *p* > 0.05.

We then explored a dataset, GSE4045, which included both conventional CRCs and CRCs with serrated morphology (Supplementary Table S2) [35]. As depicted in Figure 2a, serrated CRCs exhibited significantly higher levels of TBSS than conventional CRCs. Given that mutant *BRAF* is a defining feature of CRCs that originates from the serrated pathway, we further assembled four bulk datasets of CRC with *BRAF* mutation information: GSE39582, TCGA, Sidra-LUMC, and GSE39084. These datasets encompasses a total of 170 *BRAF*-mutant and 1140 wild-type CRCs (Supplementary Table S2) [2,36,37,38]. This large-scale analysis using multiple cohorts consistently demonstrated significantly elevated TBSS levels in *BRAF*-mutant CRCs compared to those in wild-type CRCs (Figure 2b–e). Conversely, the CCLE dataset, which comprised 57 CRC cell lines with *BRAF* mutation data [39], showed that the *BRAF* mutation status had no significant impact on the levels of TBSS, likely due to its stromal specificity (Figure 2f).

### 2.4. TGFβ-Induced Stromal Activation in Serrated Precursor Lesions

**Figure 3:**
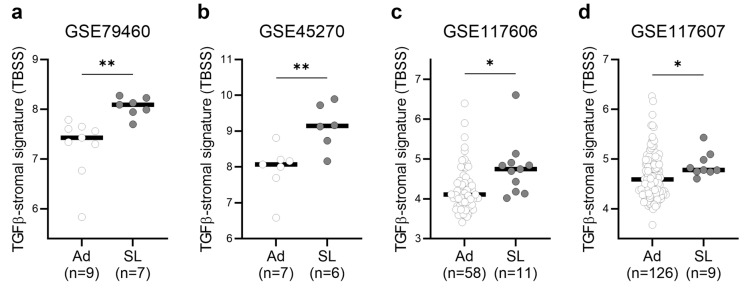
TBSS in multiple cohorts of colorectal premalignant lesions. (**a**–**d**) TBSS levels in a total of 200 conventional adenomas (Ad) and 33 serrated lesions (SL) in 4 datasets (GSE79460, GSE45270, GSE117606, and GSE117607). ** *p* < 0.01, * *p* < 0.05.

We further aimed to investigate the TGFβ-activated TME in serrated premalignant lesions. To this end, we collected four independent cohorts of colorectal precursor lesions, including GSE79460, GSE45270, GSE117606, and GSE117607 (Supplementary Table S2) [20,21,34]. In this analysis, serrated adenomas and sessile serrated adenomas, as defined in each study, were collectively considered as serrated lesions. This allowed us to examine a total of 200 conventional adenomas and 33 serrated lesions. In each of these datasets, TBSS was consistently found to be significantly increased in serrated lesions compared to conventional adenomas (Figure 3a–d).

## 3. Discussion

In the present study, we employed an in silico approach, analyzing multiple transcriptomic datasets of colorectal precursor lesions and CRCs across various platforms. To the best of our knowledge, this is the first attempt to investigate the timing and extent of TGFβ-induced stromal activation in the serrated pathway. Moreover, this is one of the largest studies comparing serrated and conventional colorectal carcinogenesis through gene expression profiling. Utilizing a multi-gene signature, namely TBSS, we inferred the levels of TGFβ-induced activation in the tumor stroma. Importantly, this signature, developed in our prior work, underwent a rigorous validation process to ascertain its specificity to the stromal component, effectively excluding transcriptional signals derived from epithelial cells. Our analyses, particularly through single-cell RNA-seq data involving about 70,000 single cells from 39 CRCs in 3 datasets, conclusively confirmed that TBSS was attributed to CAFs and endothelial cells within the TME. A subsequent evaluation across multiple bulk datasets comprised 241 conventional adenomas and 598 primary CRCs at various disease stages that consistently showed increased TGFβ-dependent stromal activation during malignant transformation and tumor progression, which was particularly evident during the transition of from conventional adenoma to carcinoma. By applying this signature to precursor lesions and CRCs with morphological or genetic features associated with serrated tumorigenesis, this study provides novel insights into the potential impact of the TGFβ-activated TME on early stages of the serrated pathway. Our findings reveal several important observations. Firstly, serrated CRCs exhibited significantly higher levels of TBSS compared to conventional CRCs. Secondly, analyses across four independent cohorts of CRCs consistently demonstrated a significant increase in TBSS in *BRAF*-mutant CRCs compared to *BRAF*-wild type CRCs. Thirdly, even in the premalignant stage, serrated precursor lesions displayed markedly elevated TBSS levels compared to conventional adenomas. This early stromal activation in serrated lesions was reproducibly observed in four independent cohorts of cancer precursor samples. While CRCs arising via the serrated pathway often lack definitive serrated morphologic features at diagnosis, serrated CRCs are presumed to arise from serrated precursors [9,40]. Similarly, *BRAF*-mutant CRCs are considered to develop from the serrated pathway, where a *BRAF* mutation typically serves as the initiating genetic event in serrated precursors, unlike conventional adenomas. Overall, our study demonstrates that both serrated CRCs and *BRAF*-mutant CRCs, which are associated with the serrated pathway, are characterized by TGFβ-activated stroma. Furthermore, our findings suggest that this stromal activation occurs earlier in premalignant lesions arising from the serrated pathway compared to those from the conventional pathway. 

In colorectal carcinogenesis, TGFβ can exert either tumor-promoting or tumor-suppressive functions, depending on the cellular context. Although TGFβ initially suppresses the growth of normal and premalignant epithelial cells, TGFβ signaling in epithelial cancer cells is known to contribute to the later stages of tumorigenesis, particularly by promoting TGFβ-induced EMT, thereby driving invasion and metastasis [15,16]. However, the influence of TGFβ in premalignant epithelial cells may differ between conventional and serrated tumorigenesis. Fessler et al. demonstrated that distinct genetic backgrounds of pre-neoplastic lesions respond differentially to TGFβ stimulation in organoid cultures [21]. Notably, conventional adenoma organoids displayed an apoptotic phenotype upon TGFβ treatment, while TGFβ can induce EMT in a *BRAF*-mutant organoid culture, an in vitro model system for serrated premalignant lesions. This is consistent with transcriptomic evidence indicating the upregulation of EMT genes in serrated precursor lesions and mesenchymal CRCs associated with poor prognosis [20]. These data suggest that epithelial TGFβ signaling is already active in serrated precursors, directing serrated precursors toward mesenchymal and poor prognosis CRCs [21]. Conversely, recent studies have emphasized the role of the TGFβ-activated tumor-promoting stroma in CRC [22,23,24,25,26]. Advanced-stage cancers often exhibit a TGFβ-rich TME enriched with CAFs producing not only the main component of the extra cellular matrix (ECM) but also various cytokines [41]. Within this tumor-promoting stroma, TGFβ secreted by CAFs and other stromal cells activates a pro-metastatic gene program, correlating with poor prognosis [15,16,23,41]. Chakravarthy et al. identified a pan-cancer transcriptional signature linked to ECM dysregulation, which was associated with cancer progression and poor prognosis [42]. Notably, cancers with high levels of this ECM signature not only exhibit activated TGFβ signaling in CAFs but also carry distinct genomic alterations, including *BRAF* and *SMAD4* mutations. Although the inactivation of TGFβ pathway genes, such as *SMAD4* mutations, is commonly associated with late-stage colorectal tumorigenesis in the conventional pathway [1,5,6], it was recently revealed that the loss of *SMAD4* or epithelial TGFβ receptor signaling accelerates the early stage initiation and progression of *BRAF*-induced serrated carcinogenesis in in vivo mouse models [12,13,14,43]. Moreover, analyses by Tong et al. involving human tumors and mouse genetic models suggested that *SMAD4* loss has a pivotal role in the transition from hyperplasia to dysplasia in early stage *BRAF*-driven serrated tumorigenesis [13]. Therefore, these studies, along with our findings, indicated that TGFβ in the TME might activate tumor epithelial cells and stromal cells in a context-dependent manner during the early stages of serrated tumorigenesis. This suggests that TGFβ might promote early serrated tumorigenesis by inducing EMT in epithelial tumor cells and/or by activating tumor-promoting stroma, potentially depending on the genetic backgrounds in premalignant lesions. In particular, loss-of-function alterations in the TGFβ pathway genes may occur early in *BRAF*-driven serrated lesions, contributing to tumor progression through the activation of tumor stroma by TGFβ. This warrants further investigation using genetically engineered in vivo models of the serrated pathway in the presence of stromal cells.

This study has several limitations inherent to its in silico, exploratory design using publicly available transcriptomic datasets. Notably, genomic alteration data are lacking for serrated precursor lesions in these databases. In many of these CRC datasets, except for one dataset containing a small number of serrated CRCs, we were not able to obtain the information on serrated adenocarcinoma. This limitation might be because adenocarcinomas arising from serrated precursors rarely exhibit definitive serrated morphology at presentation, in addition to the relatively low prevalence of serrated CRCs among all CRCs. Also, the exact source of TGFβ in serrated tumorigenesis remains uncertain.

In conclusion, our large-scale transcriptomic data analysis suggests early TGFβ-responsive stromal activation in serrated carcinogenesis, providing valuable insights into the intricate role of TGFβ in the early stages of this pathway.

## 4. Materials and Methods

### 4.1. Microarray and RNA-Seq Data Analysis

Single-cell and bulk transcriptomic datasets based on RNA-seq or Affymetrix microarray platforms used in this study are summarized in Supplementary Tables S1 and S2. We utilized the preprocessed values obtained from each dataset. If a gene was represented by multiple probes in microarrays, only the probe with the highest mean expression was used. A total of 3 single-cell RNA-seq datasets of CRCs, including GSE132465 (23 CRCs), GSE144735 (6 CRCs), and GSE146771 (10 CRCs), were downloaded from the Gene Expression Omnibus (GEO) database, accessed on 14 July 2021. A merged meta-dataset consisted of 132 conventional colorectal adenomas and 342 CRCs from 12 studies based on the Affymetrix microarray that was available from the ArrayExpress database under accession number E-MTAB-10089 accessed on 31 January 2021. We also obtained Affymetrix microarray datasets that consisted of conventional adenomas, serrated lesions, and/or CRCs, including GSE41258 (51 adenomas, 342 CRCs, and 9 CRC cell lines), GSE4045 (29 conventional CRCs and 8 serrated CRCs), GSE39582 (512 CRCs), GSE39084 (70 CRCs), GSE79460 (9 adenomas and 7 serrated lesions), GSE45270 (7 adenomas and 6 serrated lesions), GSE117606 (58 adenomas, 11 serrated lesions, and 74 CRCs), and GSE117607 (126 adenomas and 9 serrated lesions), which were downloaded from the GEO database, accessed on 7 December 2022. Furthermore, RNA-seq datasets, including The Cancer Genome Atlas (TCGA, 453 CRCs), the Sidra-LUMC (275 CRCs), and the Cancer Cell Line Encyclopedia (CCLE, 57 CRC cell lines), were obtained through cBioPortal, accessed on 30 June 2023 [44]. We also obtained somatic *BRAF* mutation data for GSE39582, GSE39084, TCGA, Sidra-LUMC, and CCLE cohorts.

In our previous studies, we identified a set of TGFβ-responsive stromal genes in CRC, including *VCAN*, *SERPINE1*, *CALD1*, *FAP*, *POSTN*, and *IGFBP7* [27,28]. Based on the expression of these six genes, we built a TGFβ-stromal signature, designated TBSS, and we computed the signature score by taking the mean expression values for each sample [28].

### 4.2. Statistical Analysis

Unpaired *t*-test with Welch’s correction was used to determine differences between two variables. For comparison across multiple groups, Welch’s one-way analysis of variance (ANOVA) was performed. All statistical analyses were conducted using GraphPad Prism v9.5.1 (GraphPad Software Inc., San Diego, CA, USA). All *p*-values were two-sided, and we considered *p*-values less than 0.05 to be statistically significant.

## Data Availability

The public datasets used and analyzed during this study are available from GEO (https://www.ncbi.nlm.nih.gov/geo/) accessed on 14 July 2021, ArrayExpress (https://www.ebi.ac.uk/biostudies/arrayexpress) accessed on 31 January 2021, and cBioPortal (https://www.cbioportal.org/) accessed on 30 June 2023.

## Supplementary Materials

The following supporting information can be downloaded at: https://www.mdpi.com/article/10.3390/ijms25094626/s1.
