# Supplementary material for: TGFβ-Responsive Stromal Activation Occurs Early in Serrated Colorectal Carcinogenesis
Source: Int J Mol Sci. 2024 Apr 24;25(9):4626. doi: 10.3390/ijms25094626 (PMC11083568; doi:10.3390/ijms25094626)
Supplement: Supplementary file 1 [file ijms-25-04626-s001.zip › ijms-2971561-supplementary.pdf]

Supplementary Table S1

Dataset	Platform		References
GSE132465	Single-cell RNA-seq	47285 single cells from 23 CRC patients	[1]
GSE144735	Single-cell RNA-seq	17678 single cells from 6 CRC patients	[1]
GSE146771	Single-cell RNA-seq	5220 single cells from 10 CRC patients	[2]

Supplementary Table S2

Dataset	Platform	Precursor			CRC				References
		Total (n=416)	Conventional	Serrated	Total (n=2014)	BRAF-WT	BRAF-Mut	Cell line	
E-MTAB-10089	Affymetrix Human Genome U133+2.0	132	132	0	342	-	-	-	[3]
GSE41258	Affymetrix Human Genome U133A	51	51	0	194	-	-	9	[4]
GSE117606	Affymetrix HT HG-U133+ PM Array Plate	69	58	11	74	-	-	-	[5]
GSE4045	Affymetrix Human Genome U133A	0	0	0	37	-	-	-	[6]
GSE39582	Affymetrix Human Genome U133+2.0	0	0	0	512	461	51	-	[7]
TCGA	RNA-seq	0	0	0	453	401	52	-	[8]
Sidra-LUMC	RNA-seq	0	0	0	275	216	59	-	[9]
GSE39084	Affymetrix Human Genome U133+2.0	0	0	0	70	62	8	-	[10]
CCLE	RNA-seq	0	0	0	57	46	11	57	[11]
GSE79460	Affymetrix HT HG-U133+ PM Array Plate	16	9	7	0	-	-	-	[12]
GSE45270	Affymetrix Human Genome U133+2.0	13	7	6	0	-	-	-	[13]
GSE117607	Affymetrix HT HG-U133+ PM Array Plate	135	126	9	0	-	-	-	[5]

References for Supplementary Tables

1. Lee, H.O.; Hong, Y.; Etlioglu, H.E.; Cho, Y.B.; Pomella, V.; Van den Bosch, B.; Vanhecke, J.; Verbandt, S.; Hong, H.; Min, J.W.; et al. Lineage-dependent gene expression programs influence the immune landscape of colorectal cancer. *Nat Genet* 2020, *52*, 594-603, doi:10.1038/s41588-020-0636-z.

2. Zhang, L.; Li, Z.; Skrzypczynska, K.M.; Fang, Q.; Zhang, W.; O'Brien, S.A.; He, Y.; Wang, L.; Zhang, Q.; Kim, A.; et al. Single-Cell Analyses Inform Mechanisms of Myeloid-Targeted Therapies in Colon Cancer. *Cell* **2020**, *181*, 442-459 e429, doi:10.1016/j.cell.2020.03.048.
3. Rohr, M.; Beardsley, J.; Nakkina, S.P.; Zhu, X.; Aljabban, J.; Hadley, D.; Altomare, D. A merged microarray meta-dataset for transcriptionally profiling colorectal neoplasm formation and progression. *Sci Data* **2021**, *8*, 214, doi:10.1038/s41597-021-00998-5.
4. Sheffer, M.; Bacolod, M.D.; Zuk, O.; Giardina, S.F.; Pincas, H.; Barany, F.; Paty, P.B.; Gerald, W.L.; Notterman, D.A.; Domany, E. Association of survival and disease progression with chromosomal instability: a genomic exploration of colorectal cancer. *Proc Natl Acad Sci U S A* **2009**, *106*, 7131-7136, doi:10.1073/pnas.0902232106.
5. Chang, K.; Willis, J.A.; Reumers, J.; Taggart, M.W.; San Lucas, F.A.; Thirumurthi, S.; Kanth, P.; Delker, D.A.; Hagedorn, C.H.; Lynch, P.M.; et al. Colorectal premalignancy is associated with consensus molecular subtypes 1 and 2. *Ann Oncol* **2018**, *29*, 2061-2067, doi:10.1093/annonc/mdy337.
6. Laiho, P.; Kokko, A.; Vanharanta, S.; Salovaara, R.; Sammalkorpi, H.; Järvinen, H.; Mecklin, J.P.; Karttunen, T.J.; Tuppurainen, K.; Davalos, V.; et al. Serrated carcinomas form a subclass of colorectal cancer with distinct molecular basis. *Oncogene* **2007**, *26*, 312-320, doi:10.1038/sj.onc.1209778.
7. Marisa, L.; de Reyniès, A.; Duval, A.; Selves, J.; Gaub, M.P.; Vescovo, L.; Etienne-Grimaldi, M.C.; Schiappa, R.; Guenot, D.; Ayadi, M.; et al. Gene expression classification of colon cancer into molecular subtypes: characterization, validation, and prognostic value. *PLoS Med* **2013**, *10*, e1001453, doi:10.1371/journal.pmed.1001453.
8. Cancer Genome Atlas, N. Comprehensive molecular characterization of human colon and rectal cancer. *Nature* **2012**, *487*, 330-337, doi:10.1038/nature11252.
9. Roelands, J.; Kuppen, P.J.K.; Ahmed, E.I.; Mall, R.; Masoodi, T.; Singh, P.; Monaco, G.; Raynaud, C.; de Miranda, N.; Ferraro, L.; et al. An integrated tumor, immune and microbiome atlas of colon cancer. *Nat Med* **2023**, *29*, 1273-1286, doi:10.1038/s41591-023-02324-5.
10. Kirzin, S.; Marisa, L.; Guimbaud, R.; De Reynies, A.; Legrain, M.; Laurent-Puig, P.; Cordelier, P.; Pradère, B.; Bonnet, D.; Meggetto, F.; et al. Sporadic early-onset colorectal cancer is a specific sub-type of cancer: a morphological, molecular and genetics study. *PLoS One* **2014**, *9*, e103159, doi:10.1371/journal.pone.0103159.
11. Ghandi, M.; Huang, F.W.; Jane-Valbuena, J.; Kryukov, G.V.; Lo, C.C.; McDonald, E.R., 3rd; Barretina, J.; Gelfand, E.T.; Bielski, C.M.; Li, H.; et al. Next-generation characterization of the Cancer Cell Line Encyclopedia. *Nature* **2019**, *569*, 503-508, doi:10.1038/s41586-019-1186-3.
12. Fessler, E.; Drost, J.; van Hooff, S.R.; Linnekamp, J.F.; Wang, X.; Jansen, M.; De Sousa, E.M.F.; Prasetyanti, P.R.; JE, I.J.; Franitza, M.; et al. TGFbeta signaling directs serrated adenomas to the mesenchymal colorectal cancer subtype. *EMBO Mol Med* **2016**, *8*, 745-760, doi:10.15252/emmm.201606184.
13. De Sousa E Melo, F.; Wang, X.; Jansen, M.; Fessler, E.; Trinh, A.; de Rooij, L.P.; de Jong, J.H.; de Boer, O.J.; van Leersum, R.; Bijlsma, M.F.; et al. Poor-prognosis colon cancer is defined by a molecularly distinct subtype and develops from serrated precursor lesions. *Nat Med* **2013**, *19*, 614-618, doi:10.1038/nm.3174.
